# Hydrogels: From Controlled Release to a New Bait Delivery for Insect Pest Management

**DOI:** 10.1093/jee/toaa183

**Published:** 2020-08-27

**Authors:** Jia-Wei Tay, Dong-Hwan Choe, Ashok Mulchandani, Michael K Rust

**Affiliations:** 1 Urban Entomology Laboratory, Department of Plant and Environmental Protection Sciences, University of Hawaii at Manoa, Honolulu, HI; 2 Department of Entomology, Riverside, CA; 3 Department of Chemical Engineering, Riverside, CA; 4 Materials Science and Engineering Program, University of California Riverside, Riverside, CA

**Keywords:** hydrogel, alginate, controlled release, ant control, pest management

## Abstract

Here, we review the literature on the development and application of hydrogel compounds for insect pest management. Researchers have used hydrogel compounds for the past few decades to achieve the controlled release of various contact insecticides, but in recent years, hydrogel compounds have also been used to absorb and deliver targeted concentrations of toxicants within a liquid bait to manage insect pests. The highly absorbent hydrogel acts as a controlled-release formulation that keeps the liquid bait available and palatable to the target pests. This review discusses the use of various types of hydrogel compounds in pest management based on different environmental settings (e.g., agricultural, urban, and natural areas), pest systems (e.g., different taxa), and modes of insecticide delivery (e.g., spray vs bait). Due to their unique physicochemical properties, hydrogel compounds have great potential to be developed into new and efficacious pest management strategies with minimal environmental impact. We will also discuss the future research and development of hydrogels in this review.

Urban pest management is crucial with over 50% of the world population living in urban areas (Neiderud 2015). In agriculture, managing pests by applying pesticides to protect crops is one of the most crucial aspects (Huang et al. 2018). According to Sharma et al. (2019), ~3.5 million tons of pesticides are applied to crops yearly worldwide (50–60% herbicides, 20–30% insecticides, and 10–20% fungicides; Pimentel 1995). However, less than 1% of these pesticides actually come in direct contact with or are consumed by target pests (Pimentel 1995, Huang et al. 2018). There are many approaches of pest management that have been developed to target the needs of these different environments. For example, controlled-release formulations provide slow and controlled release of active ingredients (AIs) commonly used in agriculture for the past few decades, reducing pesticide levels in the environment unlike traditional pesticide formulations (Roy et al. 2014, He et al. 2019). Additionally, insecticides can be formulated as a spray or combined with a phagostimulant as a bait. Thus, if the target pests can be attracted to feed on the insecticide-treated bait, it could greatly reduce the amount of insecticides needed for pest control and the amount that is left in the environment (Pimentel 1995). In these pest management approaches, hydrogels have been widely used as a matrix for pesticide delivery.

In this review, we provide a broad overview of the uses of hydrogel compounds in the controlled release of contact pesticides using microencapsulation and matrix approaches, followed by a discussion of the use of synthetic and natural hydrogel compounds to absorb and deliver the targeted concentration of AI within a liquid phagostimulant (a new baiting strategy for controlling social insect pests, such as ants and yellowjackets). The highly absorbent hydrogel acts as a controlled-release formulation that keeps the liquid bait available and palatable to the target pests. Furthermore, we explore the manufacturing methods of natural and synthetic hydrogels, followed by the advantages and disadvantages thereof. This review also considers upcoming challenges of hydrogel development for pesticide delivery and its applications.

## Controlled Release

Many hydrogel compounds have been researched as controlled-release vehicles for various AIs in agriculture. In controlled-release strategies, the insecticides are slowly delivered over time from the treated surfaces, soil, or plants in a controlled manner (Garrido et al. 2012). Some of the advantages include longer application intervals (labor-saving); stabilization of AIs against environmental degradation; and reduction in dosages, toxicity, and risks to humans (e.g., by reducing human mucous-membrane irritation) and the environment as compared with conventional pesticide sprays (Rudzinski et al. 2002, Roy et al. 2009a, b).

Controlled-release formulations have been used to deliver a broad range of insecticides, especially broad-spectrum insecticides, such as organophosphates (e.g., chlorpyrifos) and carbamate (e.g., carbaryl). These are moderately toxic chemicals widely used as agricultural insecticides in many countries (Işıklan 2007, Roy et al. 2009a). Because of their moderately high toxicity levels that cause cumulative adverse effects in both humans and nontargeted organisms, the use of chlorpyrifos, an organophosphate insecticide, has been banned in the United States for residential uses since 2002 (U.S. Environmental Protection Agency 2002) and in Yemen since 2006 (El-Zaemey et al. 2013). Chlorpyrifos is being reevaluated in some places, including the European Union, New Zealand, and the United States (e.g., restricted use in Hawaii since 2018; Natural Resources Defense Council 2018). In addition, carbaryl, a carbamate insecticide, has been banned in Europe (Pesticide Action Network 2008). Controlled-release formulations can reduce their harmful effects (He et al. 2019). In addition to contact insecticides, controlled-release formulations have been used to deliver fertilizers to plants (Sabadini et al. 2015).

There are two major approaches to incorporating an AI into a controlled-release formulation: capsules and beads. First, the AI or a core material can be encapsulated by a thin outer wall made from a natural or synthetic coating, shell, or membrane (‘capsules type’; Roy et al. 2014). It is an important industrial technique for pesticide delivery, especially in agriculture (Garrido et al. 2012). It was used to encapsulate mirex in vegetable oil which allows it to withstand weathering in controlling imported fire ants (Markin and Hill 1971). Additionally, it has been used to encapsulate other bioactive materials, such as corn stalk borer sex pheromone (Mihou et al. 2007), gypsy moth sex attractant (Cameron 1973, Richerson 1977), and microbial insecticides such as *Bacillus thuringiensis* in controlling European corn borer (Raun and Jackson 1966).

Microencapsulation is a process in which tiny droplets of liquid are coated with a continuous film of polymer to produce small capsules. It can be manufactured by various physical (e.g., spray drying, coacervation/phase separation, solvent evaporation/extraction) and chemical (e.g., interfacial polycondensation, emulsion polymerization) methods (Dubey et al. 2009). The wall can be made of hard or soft soluble material, which prevents the AI from direct exposure to the environment. Common polymeric encapsulated wall materials are gelatin, gum arabic, starch, sugar, ethyl cellulose, carboxymethyl cellulose, paraffin, polyvinyl alcohol, polyethylene, polypropylene, polystyrene, polyacrylamide, polyethers, polyesters, polyamides, polyureas, polybutadiene, polyisoprene, polysiloxanes, polyurethanes, epoxy resins, and inorganic silicates (Scher 1977, Swietoslawski et al. 2011).

A variety of combinations of synthesis methods and materials can be chosen to produce microencapsulated products for various controlled-release applications (Dubey et al. 2009). The release of the AI occurs by diffusion through the membrane barrier (Rudzinski et al. 2002). Thus, wall thickness, wall materials, wall structure, degree of penetrability, and types of AIs can be modified to manipulate the rate of the insecticide diffusion (Swietoslawski et al. 2011). The sizes of the microcapsules range from 1 μm to a few mm, depending on the application purposes (Dubey et al. 2009).

In insect pest management, microencapsulated products act as a controlled-release vehicle to regulate their release to the environment and to insects which come into contact with them at a rate that depends on the diffusion coefficient of the AI (Swietoslawski et al. 2011). When insects pass through a treated surface, contact insecticides in the microcapsules adhere to the insects’ body parts. The AI slowly diffuses through the capsule polymer membrane barrier, is absorbed by the cuticle, and penetrates the body of the insects (Stejskal et al. 2009).

Regular chemical sprays significantly contribute to volatile organic compound emissions and potentially impact air quality (CA Department of Pesticide Regulation 2020). Many volatile compounds from pesticide residues have been found in the atmosphere. A previous study showed that microencapsulated formulation of diazinon, an organophosphate insecticide (e.g., Knox Out, a household insecticide product), successfully reduced the aerial concentration of diazinon upon spray treatment as compared with its emulsifiable concentrate formulation (Tsuji 2001). Microcapsules are a safer formulation than emulsifiable concentrate and wettable powders for delivering insecticides with contact activity. Insecticides formulated into this ‘capsules type’ have reduced odor, increased bioavailability, improved stability, and pose a lower risk to pesticide applicators (Stejskal et al. 2009). One of the factors that affects the efficacy of insecticides is their repellency to target pests (Knight and Rust 1990, Tabaru et al. 2001, Tay and Lee 2015a). A previous study shows that the repellency of organophosphate insecticides such as diazinon and fenitrothion may also be overcome by microencapsulation (Tabaru et al. 2001).

In addition to the encapsulation technique, the AI can be completely integrated into a matrix (‘beads type’) to achieve slow release. The ‘beads type’ formulation is produced via internal gelation without an outer wall. Gelation proceeds from the outer surface to the center of the bead and the AI is dissolved and dispersed in many small cells in a continuous phase of polymer matrix (Roy et al. 2014). Swellable polymeric beads, which involve the integration of water into the polymer matrix, are often used in this type of controlled delivery system (Roy et al. 2009a, b; 2014).

Swellable matrices’ sensitivity to water contribute to their highly absorbent property. They have the ability to absorb water many times their dry mass and significantly expand in their volume without undergoing dissolution (Ahmed 2015). Therefore, they have the potential to be used as delivery vehicles not only for the controlled release of contact insecticides but also for liquid baits. Their porosity also permits absorption of various conditioning liquids to deliver wide ranges of water-soluble AIs. This approach has also been used to develop new baiting methods for ants. For example, after producing the swellable polymers, they can be immersed in a conditioning liquid containing AIs and a sucrose solution, resulting in a significant expansion of their volumes. The penetration of the conditioning liquid allows the AI to diffuse through the swollen polymer matrix along with the sucrose solution (phagostimulant) to be slowly delivered to foraging insects such as pest ants (Rust et al. 2015).

In the case of liquid ant baits, the bait solutions are removed by these insects imbibing them from the surface of the matrices, and the matrices are not removed or consumed (Rust et al. 2015). The saturated beads individually act as micro-sized controlled-release liquid bait stations, dispensers, and reservoirs. Although they lose water over time, they re-hydrate in response to the presence of water and resume their insecticidal activity. Compared with the ‘capsules type’, these unique properties of the polymer matrix (‘beads type’) make it a novel candidate to deliver both liquid baits and insecticides to insects.

## Hydrogels

For the ‘beads type’ controlled-release system, hydrogels are a popular polymeric matrix. Hydrogels are defined as two- or multi-component systems consisting of a three-dimensional network structure with the ability to absorb a large volume of water and expand their volume without being dissolved (Işıklan 2007, Ahmed 2015). They can be made from natural or synthetic polymers, or combinations of the two (He et al. 2019). Synthetic hydrogels are usually acrylate- and polyacrylamide-based while natural hydrogels are made of alginates, carboxymethyl cellulose, chitosan, pectin, or other products (Ahmed 2015). Hydrogels hydrate to different degrees because of the presence of different hydrophilic functional groups on the backbone of the polymer (i.e., –OH, –CONH_2_, etc.) and different polymer compositions (Rudzinski et al. 2002, Ahmed 2015) and they are water insoluble because of the crosslinks between the network chains (Ahmed 2015). The degree of hydration also depends on temperature, pH, and pressure (Aouada et al. 2010).

Hydrogels were developed initially for used in biomedical and healthcare applications, such as drug delivery (Tønnesen and Karlsen 2002, Wang et al. 2010), scaffolds for tissue engineering of bone and cartilage (El-Sherbiny and Yacoub 2013), and wound healing (Saarai et al. 2011, Zhu et al. 2019). The versatility of hydrogels allows their controlled-release applications in various fields, such as in agriculture to deliver pesticides and fertilizers (Aouada et al. 2010).

In the past, traditional synthetic hydrogels made from acrylic (acrylate-based) and polyacrylamide were used in agriculture (Montesano et al. 2015). However, they are not biodegradable and are potential soil pollutants. Furthermore, in agricultural applications, the gradual degradation of synthetic polymers may adversely affect soil fertility (Roy et al. 2014). Due to the increasing attention of environmental issues, biodegradable hydrogels have become preferred over the synthetic ones in some situations. Renewability, biocompatibility, and nontoxicity of biodegradable hydrogels make them an attractive option for agricultural applications, such as retaining moisture (Montesano et al. 2015) and controlling insect pests (Rudzinski et al. 2002, He et al. 2019). However, Ahmed (2015) also argued that natural hydrogels have been gradually replaced by synthetic hydrogels due to the higher water absorption capacity and gel strength of the latter.

## Hydrogel Baits

More than $1.7 billion are spent annually to hire pest management professionals (PMPs) for urban ant control in the United States (Curl 2005). Ants also tend honeydew-producing hemipteran pests (aphids, mealybugs, and scale insects) in agriculture (Moreno et al. 1987, Calabuig et al. 2015) and displace native insects, impacting the ecology and economics of natural settings (Lee et al. 2017). In urban environments, management of ants often relies heavily on various insecticidal sprays (Knight and Rust 1990). Consequently, these insecticides, such as fipronil and various pyrethroids, are frequently detected in urban waterways, threatening the quality of ground and surface water, soil, and air (Weston et al. 2009; Lao et al. 2010; Greenberg et al. 2010, 2014, 2017; Delgado-Moreno et al. 2011; Gan et al. 2012). Frequent detection of these AIs is a concern because of the potential effects on nontarget organisms and ecosystems (Bonmatin et al. 2015).

A proven method to reduce pesticide runoff is baiting (Klotz et al. 2003, 2009; Nelson and Daane 2007; Cooper et al. 2008; Greenberg et al. 2013). Liquid baiting (phagostimulant formulated with slow-acting AIs) has been shown to be an effective alternative to insecticidal sprays in controlling several sugar-feeding ant species (Rust et al. 2004). Baits exploit the recruitment and food sharing behavior of ants so that the AI can be spread to all the colony members, including the queens via trophallaxis (Oi et al. 2000; Rust et al. 2004; Suiter et al. 2006; Tay and Lee 2014, 2015b; Tay et al. 2014; Welzel and Choe 2016). The current liquid baiting method requires bait stations to store and dispense the sucrose bait (Hogg et al. 2018). Although numerous new bait stations designs have been developed and registered in the market, bait stations are typically expensive and labor-intensive to maintain (Nelson and Daane 2007, Rust et al. 2015, Cooper et al. 2019). These limitations prevent baiting from being widely adopted by PMPs and farmers in urban and agricultural settings (Rust et al. 2015, Cooper et al. 2019).

To overcome the limitations of insecticide spraying and conventional liquid baiting, research has been done using synthetic and natural hydrogels to deliver liquid bait to ants without using commercial bait stations or dispensers (Boser et al. 2014; Buczkowski et al. 2014a, b; Rust et al. 2015; Tay et al. 2017; Cooper et al. 2019). Hydrogels can be used as controlled-release vehicles because they keep the liquid sucrose bait palatable for an extended period by retaining water (Buczkowski et al. 2014a, b; Rust et al. 2015). Hydrogels are inexpensive, effective, and low maintenance, which makes them suitable for sustainable integrated pest management programs. When ants feed on the liquid from the surface of the hydrogel baits, they ingest the liquid bait but do not normally consume the hydrogel matrix. The liquid bait is then shared with the other colony members. The AI must be formulated at a concentration that has a delayed toxic effect to allow the complete spread of the AI among colony members (Rust et al. 2004).

Although the use of hydrogels to deliver liquid baits is lesser known as compared to the use of hydrogels for delivery of contact insecticide, several papers reported the use of baits prepared from commercially available synthetic polyacrylamide hydrogel in recent years (2014–2020). In these studies, either spherical (Deco Beads, RM Chemical, Cleveland, OH) or irregularly shaped water-storing polyacrylamide hydrogels (Miracle-Gro Lawn Products, Inc., Marysville, OH) were used to deliver liquid bait targeting Argentine ants, *Linepithema humile* (Mayr) (Buczkowski et al. 2014a, Rust et al. 2015, Cooper et al. 2019), yellow crazy ants, *Anoplolepis gracilipes* (Fr. Smith) (Peck et al. 2017), and western yellowjackets, *Vespula pensylvanica* (Saussure) (Rust et al. 2017, Choe et al. 2018). Polyacrylamide hydrogels infused with tiny amounts of thiamethoxam or boric acid effectively reduced and managed invasive Argentine ants in a commercial plum orchard in South Africa (Buczkowski et al. 2014b), California vineyards (Cooper et al. 2019), and ecologically sensitive areas of the California Channel Islands (Boser et al. 2014, Rust et al. 2015, Merrill et al. 2018). Hydrogel baits containing dinotefuran also effectively managed invasive yellow crazy ants at Johnston Atoll (Peck et al. 2015, 2017).

In the conservation areas of the California Channel Islands (Santa Cruz and San Clemente Islands), polyacrylamide hydrogel baits containing sucrose solution and insecticide thiamethoxam were spread by hand, farm fertilizer spreaders, and all-terrain vehicles with a motorized hopper and larger amounts (hundreds of kilograms) of the hydrogel bait were scattered from aircraft to rugged and inaccessible natural areas (Boser et al. 2014, 2017; Rust et al. 2015; Merrill et al. 2018). These treatments effectively reduced Argentine ant activities over 74 ha (Boser et al. 2017) and 177 ha (Merrill et al. 2018) and were able to be applied in rugged terrain or dense vegetation, where placing and maintaining numerous liquid bait stations was unfeasible. Only arthropods were observed to visit the hydrogel baits. Of these, 94.1% were ants and 5.9% were isopods and other abundant and nonsensitive arthropods (Boser et al. 2014) while only 3% of all arthropod visits to hydrogels were pollinating insects (Buczkowski 2020).

**Fig. 1.**
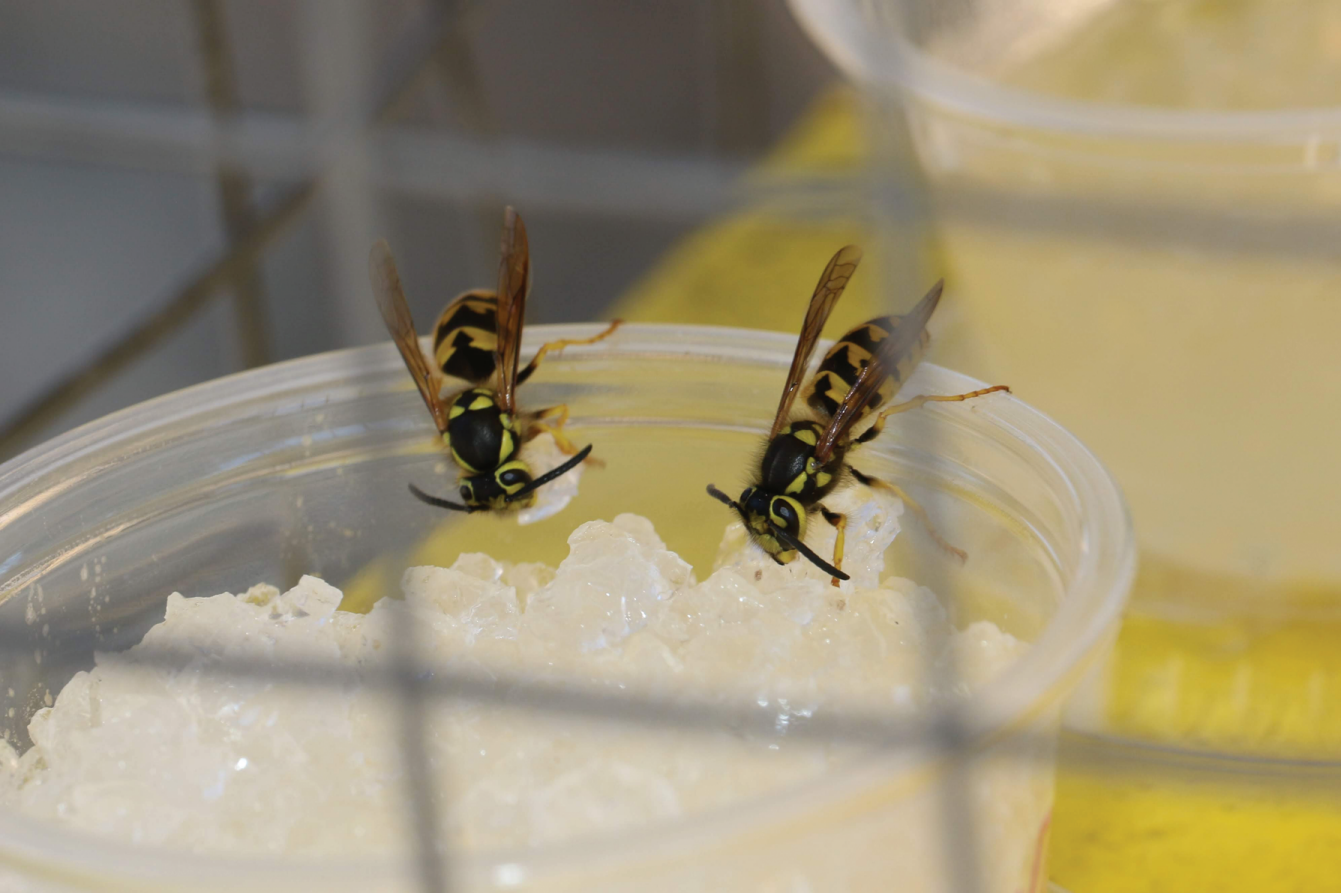
Yellowjackets carry small pieces of polyacrylamide hydrogel baits hydrated in chicken juice containing an AI, back to the nest.

Using a different AI (fipronil) and phagostimulant (chicken juice), other papers reported the use of polyacrylamide hydrogel baits in field cages hung on trees to control western yellowjackets in urban recreational areas (Rust et al. 2017, Choe et al. 2018) (Fig. 1). Yellowjackets pose a serious human health concern due to their habit of scavenging human food. Integrating insecticides into their preferred food, such as fresh meats, has been a challenge making yellowjacket baiting more difficult to perform. These baits tend to lose moisture quickly and yellowjacket foragers refuse to carry them back to the nest. Baits with water-storing polyacrylamide hydrogels retained moisture of the chicken juice and were readily retrieved by foragers and provided approximately a 74–96% reduction in the foraging activity of yellowjackets (Choe et al. 2018). Previous studies suggested that polyacrylamide hydrogels have the potential for delivering liquid baits with various phagostimulants and AIs to these target insect pests effectively using different approaches without significant effects on nontarget organisms and the ecosystem (Boser et al. 2014, Buczkowski 2020).

## Safety of Synthetic Polyacrylamide Versus Natural Alginate Hydrogels

Synthetic polyacrylamide hydrogels have higher water absorption capacity and gel strength as compared with naturally occurring polymers such as protein (i.e., collagen, gelatin) and polysaccharides (i.e., starch, alginate) (Ahmed 2015). However, upon exposure to certain environmental conditions, such as a temperature >35°C, cross-linked polyacrylamide hydrogel slowly degrades into its monomer, acrylamide (Holliman et al. 2005), which is listed as a toxic chemical, a cumulative neurotoxin, and a potential carcinogen by the World Health Organization (WHO 1985, International Agency for Research on Cancer 1994).

Natural, biodegradable hydrogels may be safer alternatives to synthetic hydrogels. Among other natural polymers, alginates are widely used as carriers in the controlled release of pesticides for agricultural applications due to their biodegradability and exceptional gelling properties, which allow easy gel formation without needing heat (Roy et al. 2009a). Sodium alginate (Na-Alg) is produced by algae and bacteria and is a gum extracted from the cell wall of fast-growing brown algae. It is an abundant polysaccharide consisting of (1–4)-linked β-d-mannuronic acid (M) and α-l-guluronic acid (G) monomers of varying proportions and sequences, which determine the physical properties of the alginate hydrogels (Murata et al. 2004). Due to its biodegradability, alginate hydrogels may degrade in the field (i.e., on the soil) in a few weeks after applications, without accumulating in the environment, as compared to polyacrylamide hydrogels that may remain on the soil for months. Additionally, if used for the delivery of fertilizers or contact pesticides, the decomposition of alginate hydrogel itself may enhance fertility of plants (Roy et al. 2014).

Studies have indicated that the use of natural hydrogels can reduce pesticide exposure to the environment. Kulkarni et al. (2000, 2002) investigated the release of a natural pesticide, neem (*Azadirachta indica* A. Juss.) seed oil and chlorpyrifos by crosslinking Na-Alg with glutaraldehyde. Işıklan (2007) prepared carbaryl-loaded calcium alginate and nickel alginate hydrogel beads with calcium chloride solution (CaCl_2_) and nickel (II) chloride solution (NiCl_2_). Roy et al. (2009b) prepared cypermethrin-loaded calcium alginate-gelatin beads with CaCl_2_. Roy et al. (2009a) also investigated the release of chlorpyrifos by crosslinking Na-Alg and starch with CaCl_2._ In addition to alginate, Işıklan (2004) investigated the release of carbaryl by crosslinking sodium carboxymethylcellulose (NaCMC) with copper (II) chloride (CuCl_2_). Most of these studies used two valance ions such as calcium from CaCl_2_, copper from CuCl_2_, or nickel from NiCl_2_, which replace the sodium ions in the Na-Alg solution to form a solid calcium/copper/nickel alginate hydrogel. The above studies provide a common understanding of the use of alginates and other natural hydrogel for the controlled release of pesticides in agriculture.

## Alginate as an Ant Bait

Apart from using natural hydrogel to deliver contact insecticides, our research group has proposed the potential of alginate hydrogel as an alternative matrix for absorbing and delivering liquid sucrose baits to ants (Tay et al. 2017). Using 0.0001% thiamethoxam as an AI, the efficacy results from the laboratory and urban field settings (i.e., 79% reduction in ant activities; estimated by consumption of a sucrose solution in monitoring vials over 24 h) showed good promise and support the use of alginate hydrogel baits for larger area-wide applications (Tay et al. 2017).

**Fig. 2.**
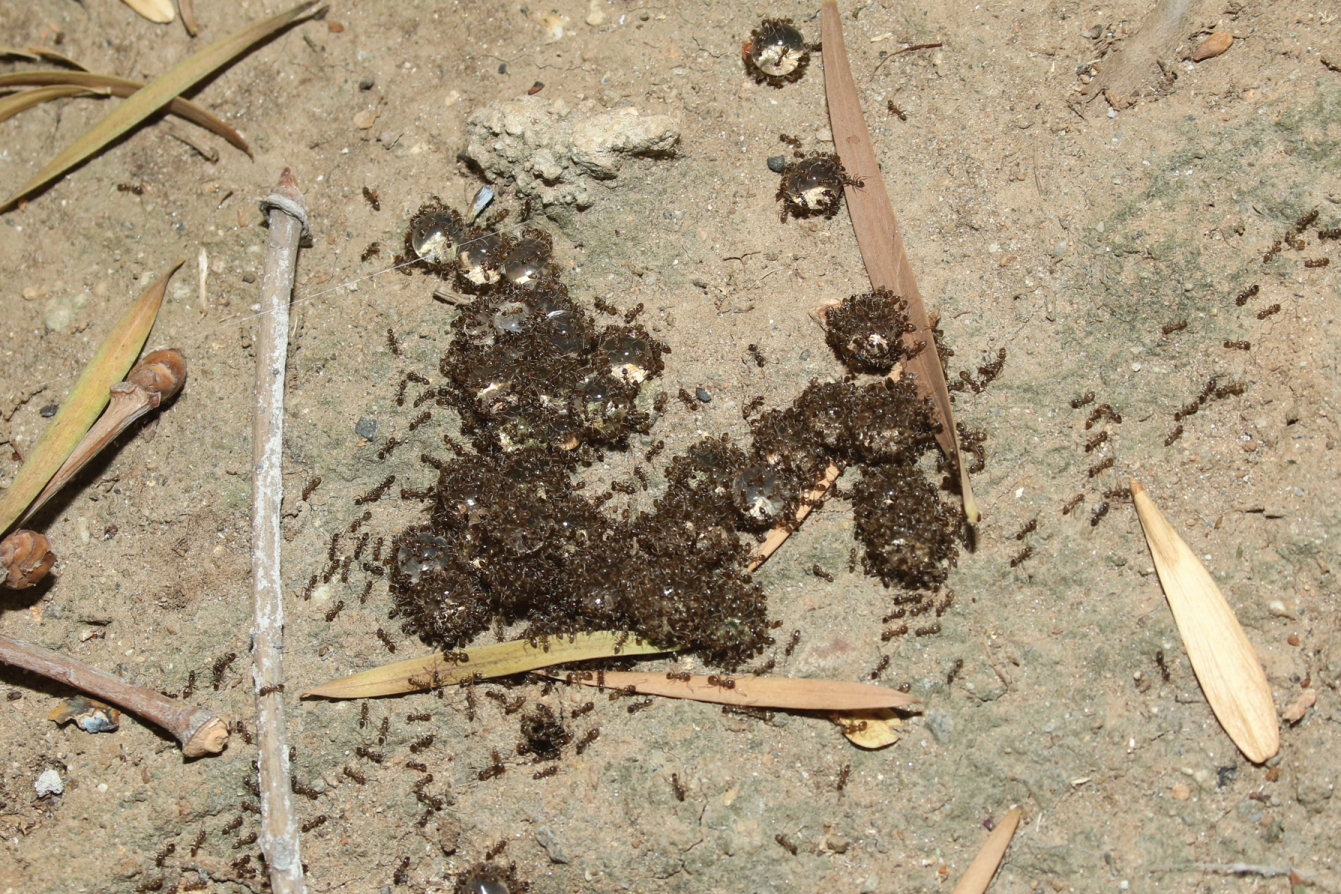
Argentine ants create trails to the alginate hydrogels containing liquid sucrose baits and continue to swarm the food source.

Since the recent introduction of the protocol developed by Tay et al. (2017) for engineering alginate hydrogel as a bait matrix, they have been adopted and used to manage field populations of invasive ants in urban, agricultural, and natural environments in the Hawaiian Islands (Krushelnycky 2019) and California (Schall et al. 2018, McCalla et al. 2020). For example, an experimental study reported that both natural (e.g., textured vegetable protein and alginate) and synthetic (e.g., polyacrylamide) hydrogels incorporated with 0.0005% thiamethoxam, 0.05% indoxacarb, or 0.005% dinotefuran, successfully controlled Argentine ant and yellow crazy ant populations in the natural areas of the Hawaiian Islands (Krushelnycky 2019). Additionally, an ongoing study investigating the efficacies of alginate hydrogels impregnated with dinotefuran has shown promising results toward managing the tawny crazy ant, *Nylanderia fulva* in Florida (D.H.O., unpublished data). Other field studies showed that alginate hydrogel baits incorporated with 0.0001% thiamethoxam provided an effective and sustainable alternative treatment option to chlorpyrifos spray programs in California citrus for Argentine ant control (Schall et al. 2018, McCalla et al. 2020). In the field, most alginate baits were retrieved by invasive ants that continue to swarm them, protecting the food source from other arthropods (Fig. 2).

## Manufacture of Hydrogel Baits

Most of the commercialized polymers are manufactured by polymerization of formulation of monomer with initiators and additives using batch or semibatch reactors in bulk, solution, or suspension at a given temperature (Ahmed 2015). Recently, the inverse-suspension polymerization technique has been widely used to mass produce polyacrylamide hydrogels from the acrylamide subunits where an aqueous solution of the acrylamide is suspended in an organic phase and initiators are added to polymerize the mixture (Rudzinski et al. 2002, Ahmed 2015). The newly formed polyacrylamide hydrogels are then washed to remove monomers, crosslinking agents, and initiators. This technique has been commonly used to produce polyacrylamide hydrogels due to its easy removal of the hazardous, residual acrylamide monomers in the polymer (Ahmed 2015).

Synthetic and natural hydrogels can be synthesized by commonly used methods in polymer chemistry (Rudzinski et al. 2002). Currently, the procedures to scale up hydrogel productions are well developed in industrial application for biotechnology, such as cell entrapment in hydrogels for immobilization (Matulovic et al. 1986). However, the procedures to mass produce hydrogel baits for insecticide delivery have yet to be developed. Tay et al. (2017) explored the use of modified shower head nozzles to mass produce spherically shaped alginate hydrogel baits. In the presence of calcium ions in the crosslinking solution, Na-Alg droplets can form distinct hydrogel beads immediately at room temperature (Leong et al. 2016). CaCl_2_ is the most commonly used salt for this purpose because it is nontoxic and readily soluble in water. During the process of crosslinking, calcium ions from the CaCl_2_ replace the sodium ions from the Na-Alg solution, forming solid calcium alginate hydrogels instantly.

To produce spherical and similarly shaped hydrogels, the distance between the nozzles and the surface of CaCl_2_ solution was optimized. In addition, the flow rate of Na-Alg is also crucial to make spherically shaped hydrogels. The viscosity of the alginate and the nozzle size may affect the flow rate (Tay et al. 2017). If the flow rate was too high due to low viscosity of the alginate or large nozzle size, a string of alginate was produced rather than discrete spherically shaped hydrogels. It was also important to stir the crosslinking solution in the process of making hydrogels to prevent the beads from adhering to each other (Tay et al. 2017, McCalla et al. 2020).

For baiting ants, blank alginate hydrogel beads were sieved from the crosslinking solution and subsequently allowed to swell in a 25% sucrose solution that sugar-feeding ants prefer (Klotz and Shorey 2000) along with a known concentration of the AI. Blank alginate hydrogel beads were allowed to condition in the liquid bait for 24 h to ensure that equilibrium has achieved and those compounds in the conditioning liquid, with a known concentration, were incorporated into the final (fully hydrated) baits (Tay et al. 2017). An enzyme-linked immunosorbent assay (ELISA) confirmed that the AI added to sucrose solution was absorbed and penetrated the hydrogel beads (Tay et al. 2017).

In addition to spherically shaped hydrogels, alginate hydrogels can also be prepared in other shapes such as thin films (Ahmed 2015). For example, calcium alginate hydrogels can be formed by immersion of 2% Na-Alg in a 5% CaCl_2_ solution while stirring (Cardea et al. 2013). Due to the versatility of hydrogels, their concentrations and preparation methods can be tailored to produce hydrogels of various sizes, strength, absorptivity, entrapment efficiency, and rate of release to meet the needs of different applications in various fields (Smidsrod and Draget 1996, Roy et al. 2014).

Several improvements in alginate hydrogel design are necessary for them to be effectively developed as a bait. After the crosslinking process, wet or fresh alginate hydrogel swells in a conditioning liquid (e.g., sucrose solution). However, they lose, in part, the ability to swell in the conditioning liquid if they are dried in the air for several hours before the conditioning process, unlike commercially available polyacrylamide hydrogel. If wet hydrogel beads are swelled in the conditioning liquid, the amount of water initially contained in the preconditioned beads should be considered when making the conditioning liquid, in order to achieve desirable concentrations in the hydrated beads (Tay et al. 2017). To ensure that optimal concentration of sucrose solution is achieved, the concentrations of sucrose solution may be checked using a refractometer during the conditioning process. Both natural and synthetic hydrogels hydrated with sucrose solution must be stored in a refrigerator to prevent fermentation, which could affect their attractiveness toward ants.

## Future Directions on Hydrogel Baits for Pest Management

Previous studies show that hydrogel baits lose their attractiveness to ants when they lose more than 50% of their water through desiccation (Rust et al. 2015, Tay et al. 2017). Although water loss rates of alginate hydrogels reported in Tay et al. (2017) were comparable with that of synthetic polyacrylamide hydrogels reported in Buczkowski et al. (2014a), hydrogels generally have rapid water loss under hot, dry field conditions, especially when they are exposed to dry soil. However, the water loss rates of the hydrogels are dependent upon the humidity in the environment and the moisture in the substrate (Tay et al. 2017). Hydrogels are capable of absorbing water from a moist substrate, which compensates for water loss through surface evaporation. Hydrogels can be rehydrated via irrigation or rainfall and the rehydration process allows the hydrogels to attract ants again. Future studies should focus on improving the water retainability of the hydrogel beads. This aspect warrants further study.

In the case of Argentine ants, their synthetic trail pheromone, (Z)-9-hexadecenal, added to the hydrogel baits improved the initial discovery and consumption of the bait by the foraging ants (Welzel and Choe 2016) before the hydrogels lost too much moisture (McCalla et al. 2020). Once the ants discover the hydrogel baits, they create their own trail to the hydrogel baits that their nestmates will follow (Fig. 2). The addition of a pheromone could further improve target specificity of the bait.

The concentrations of the AI used in all the previous hydrogel studies were extremely low as compared with those of sprays (Buczkowski et al. 2014a, b; Boser et al. 2014, 2017; Rust et al. 2015; Tay et al. 2017; Merrill et al. 2018). Future studies will examine natural hydrogels with other lower risk insecticides such as insect growth regulators or natural insecticides for pest management, especially in the urban environment where children and pets are present.

High water solubility, effectiveness in wide ranges of concentrations, and lack of repellency in an AI are important properties for formulating an efficacious sweet liquid bait (Rust et al. 2004). However, if an AI is not readily soluble in water, it can be emulsified into water with emulsifier or solvent. In addition to sucrose solution or chicken juice, the conditioning liquid can also be substituted with other preferred phagostimulants or attractants. As a result, it might be used on wider range of pests other than ants and yellowjackets. These aspects need further investigation.

For commercial development and application, it will also be important to develop methods for packaging the hydrogels. For example, the bait needs to be packaged in a container so that the application and storage will be user-friendly and safe. To increase the shelf life of the baits, preservatives will need to be tested to prevent the spoilage of the baits, while not compromising the bait uptake by the pest insects. For example, sodium benzoate at 0.25% could be incorporated in liquid baits as preservatives. Manufacturing, storage, and transport of hydrogels in a dry form is a potential cost-effective, user-friendly, and feasible method. Dry hydrogels could be hydrated and ‘activated’ by consumers immediately before the application. This approach will minimize the risk of spoilage, spill, and reduce the weight and cost during shipping and storage.

In natural environments, it is not feasible to maintain and service liquid baits in bait stations to control invasive ants. In agricultural areas, hydrogels can be applied on the soil around tree trunks where ants trail and forage, in order to manage the ant populations that interferes with biological control. It would be important to know the ecology of the target insects to improve its utility in the field. For example, knowing their foraging patterns, population size, the time and seasons of application, and the presence of alternative food sources helps in the development of optimal hydrogel baits application rates to achieve effective pest control with minimal materials and costs. Future tests should also aim to determine the efficacy of natural hydrogel baits in a larger scale in urban, agricultural, or natural environments.

## Summary

Controlled-release strategies have been extensively researched as a mean of applying insecticides with minimal contamination. As a matrix to achieve the controlled release, hydrogels have been widely used to deliver pesticides for the past few decades. Moreover, hydrogel compounds have been recently introduced as bait matrices that combine a nonrepellent AI with attractive liquid food substances to exploit insect foraging and trophallaxis behaviors to effectively control some social insect populations. Several empirical studies have shown that the use of hydrogels provide a highly efficient and low-impact outdoor baiting option for ants, yellowjackets, and other pests. Incorporation of target insect pheromones can further increase the target specificity. Due to its minimal impact on the environment and high efficacy in controlling insect pests, the development and application of hydrogel-based baits represent new directions for integrated pest management programs. We expect that alginate hydrogel baits will be commercialized by interested manufacturers for wide-scale use in various environmental settings. Future studies on the environmental safety will be required in order to register hydrogel baits as a tool for insect pest management. Future investigations will need to determine whether natural hydrogels are able to compete with synthetic ones, in terms of cost and performance.

## References

[CIT0001] Ahmed E M 2015 Hydrogel: preparation, characterization, and applications: a review. J. Adv. Res. 6: 105–121. 25750745 10.1016/j.jare.2013.07.006 PMC4348459

[CIT0002] Aouada F A, de Moura M R, Orts W J, and Mattoso L H C 2010 Polyacrylamide and methylcellulose hydrogel as delivery vehicle for the controlled release of paraquat pesticide. J. Mater. Sci. 45: 4977–4985.

[CIT0003] Bonmatin J M, Giorio C, Girolami V, Goulson D, Kreutzweiser D P, Krupke C, Liess M, Long E, Marzaro M, Mitchell E A, et al. 2015 Environmental fate and exposure; neonicotinoids and fipronil. Environ. Sci. Pollut. Res. Int. 22: 35–67. 25096486 10.1007/s11356-014-3332-7 PMC4284396

[CIT0004] Boser C L, Hanna C, Faulkner K R, Cory C, Randall J M, and Morrison S A 2014 Argentine ant management in conservation areas: results of a pilot study. Monogr. West. N. Am. Nat. 7: 518–530.

[CIT0005] Boser C L, Hanna C, Holway D A, Faulkner K R, Naughton I, Merrill K, Randall J M, Cory C, Choe D H, and Morrison S A 2017 Protocols for Argentine ant eradication in conservation areas. J. Appl. Entomol. 141: 540–550.

[CIT0006] Buczkowski G 2020 Hydrogel baits pose minimal risk to non-target insects and beneficial species. Entomol. Exp. Appl. In press.

[CIT0007] Buczkowski G, Roper E, and Chin D 2014a Polyacrylamide hydrogels: an effective tool for delivering liquid baits to pest ants (Hymenoptera: Formicidae). J. Econ. Entomol. 107: 748–757. 24772557 10.1603/ec13508

[CIT0008] Buczkowski G, Roper E, Chin D, Mothapo N, and Wossler T 2014b Hydrogel baits with low-dose thiamethoxam for sustainable Argentine ant management in commercial orchards. Entomol. Exp. Appl. 153: 183–190.

[CIT0009] (CA DPR) CA Department of Pesticide Regulation 2020 Nonfumigant volatile organic compound (VOC) regulations product list (updated 6 March 2020). Available from http://www.cdpr.ca.gov/docs/emon/vocs/vocproj/nonfum_voc_prod_list.pdf.

[CIT0010] Calabuig A, Tena A, Wäckers F L, Fernández-Arrojo L, Plou F J, Garcia-Marí F, and Pekas A 2015 Ants impact the energy reserves of natural enemies through the shared honeydew exploitation. Ecol. Entomol. 40: 687–695.

[CIT0011] Cameron E A 1973 Disparlure: a potential tool for gypsy moth population manipulation. Bull. Entomol. Soc. Am. 19: 15–19.

[CIT0012] Cardea S, Baldino L, De Marco I, Pisanti P, and Reverchon E 2013 Supercritical gel drying of polymeric hydrogels for tissue engineering applications. Chem. Eng. Trans. 32: 1123–1128.

[CIT0013] Choe D H, Campbell K, Hoddle M S, Kabashima J, Dimson M, and Rust M K 2018 Evaluation of a hydrogel matrix for baiting western yellowjacket (Vespidae: Hymenoptera). J. Econ. Entomol. 111: 1799–1805. 29850868 10.1093/jee/toy139

[CIT0014] Cooper M L, Daane K M, Nelson E H, Varela L G, Battany M C, Tsutsui N D, and Rust M K 2008 Liquid baits control Argentine ants sustainably in coastal vineyards. Calif. Agric. 62: 177–183.

[CIT0015] Cooper M L, Hobbs M B, Boser C L, and Varela L G 2019 Argentine ant management: using toxin-laced polyacrylamide crystals to target ant colonies in vineyards. Catalyst Discovery Pract. 3: 23–30.

[CIT0016] Curl G 2005 A strategic analysis of the U.S. structural pest control industry-the 2005 season: a survey of PMP’s in the U.S. Gary Curl Specialty Products Consultants. LLC, Mendham, NJ.

[CIT0017] Delgado-Moreno L, Lin K, Veiga-Nascimento R, and Gan J 2011 Occurrence and toxicity of three classes of insecticides in water and sediment in two Southern California coastal watersheds. J. Agric. Food Chem. 59: 9448–9456. 21819079 10.1021/jf202049s

[CIT0018] Dubey R, Shami T C, and Rao K U B 2009 Microencapsulation technology and applications. Def. Sci. J. 59: 82–95.

[CIT0019] El-Sherbiny I M, and Yacoub M H 2013 Hydrogel scaffolds for tissue engineering: progress and challenges. Glob. Cardiol. Sci. Pract. 2013: 316–342. 24689032 10.5339/gcsp.2013.38 PMC3963751

[CIT0020] El-Zaemey S, Fritschi L, and Heyworth J 2013 Occupational pesticide exposure among Yemeni women. Environ. Res. 122: 45–51. 23332892 10.1016/j.envres.2012.12.002

[CIT0021] Gan J, Bondarenko S, Oki L, Haver D, and Li J X 2012 Occurrence of fipronil and its biologically active derivatives in urban residential runoff. Environ. Sci. Technol. 46: 1489–1495. 22242791 10.1021/es202904x

[CIT0022] Garrido E M, Santos M, Silva P, Cagide F, Garrido J, and Borges F 2012 Host-guest complexes of phenoxy alkyl acid herbicides and cyclodextrins. MCPA and beta-cyclodextrin. J. Environ. Sci. Health B 47: 869–875.

[CIT0023] Greenberg L, Rust M K, Klotz J H, Haver D, Kabashima J N, Bondarenko S, and Gan J 2010 Impact of ant control technologies on insecticide runoff and efficacy. Pest Manag. Sci. 66: 980–987. 20730990 10.1002/ps.1970

[CIT0024] Greenberg L, Tollerup K E, and Rust M K 2013 Control of Argentine ants (Hymenoptera: Formicidae) in citrus using methoprene and imidacloprid delivered in liquid bait stations. Fla. Entomol. 96: 1023–1029.

[CIT0025] Greenberg L, Rust M K, Richards J, Wu X, Kabashima J, Wilen C, Gan J, and Choe D H 2014 Practical pest management strategies to reduce pesticide runoff for Argentine ant (Hymenoptera: Formicidae) control. J. Econ. Entomol. 107: 2147–2153. 26470080 10.1603/EC14097

[CIT0026] Greenberg L, Rust M K, Wright S, and Choe D H 2017 Argentine ant control around homes: efficacy of treatments and urban runoff. Int. J. Pest Manage. 63: 242–250.

[CIT0027] He F, Zhou Q, Wang L, Yu G, Li J, and Feng Y 2019 Fabrication of a sustained release delivery system for pesticides using interpenetrating polyacrylamide/alginate/montmorillonite nanocomposite hydrogels. Appl. Clay Sci. 183: 105347.

[CIT0028] Hogg B N, Nelson E H, Hagler J R, and Daane K M 2018 Foraging distance of the Argentine ant in California Vineyards. J. Econ. Entomol. 111: 672–679. 29361129 10.1093/jee/tox366

[CIT0029] Holliman P J, Clark J A, Williamson J C, and Jones D L 2005 Model and field studies of the degradation of cross-linked polyacrylamide gels used during the revegetation of slate waste. Sci. Total Environ. 336: 13–24. 15589246 10.1016/j.scitotenv.2004.06.006

[CIT0030] Huang B, Chen F, Shen Y, Qian K, Wang Y, Sun C, Zhao X, Cui B, Gao F, and Zeng Z 2018 Advances in targeted pesticides with environmentally responsive controlled release by nanotechnology. Nanomaterials 8: 102. 10.3390/nano8020102 PMC5853733 29439498

[CIT0031] (IARC) International Agency for Research on Cancer 1994 Acrylamide, pp. 389–433. *In* IARC monographs on the evaluation of the carcinogenic risk of chemicals to humans. IARC, Lyon, France.

[CIT0032] Işıklan N 2004 Controlled release of insecticide carbaryl from crosslinked carboxymethylcellulose beads. Fresenius Environ. Bull. 13: 537–544.

[CIT0033] Işıklan N 2007 Controlled release study of carbaryl insecticide from calcium alginate and nickel alginate hydrogel beads. J. Appl. Polym. Sci. 105: 718–725.

[CIT0034] Klotz J H, and Shorey H H 2000 Low-toxic control of Argentine ants using pheromone-enhanced liquid baits. California Department of Consumer Affairs; CDCA 84SA8020-07.

[CIT0035] Klotz J H, Rust M K, Gonzalez D, Greenberg L, Costa H, Phillips P, Gispert C, Reierson D A, and Kido. K 2003 Directed sprays and liquid baits to manage ants in vineyards and citrus groves. J. Agric. Urban Entomol. 20: 31–40.

[CIT0036] Klotz J H, Rust M K, Field H C, Greenberg L, and Kupfer K 2009 Low impact directed sprays and liquid baits to control Argentine ants (Hymenoptera: Formicidae). Sociobiology 54: 101–108.

[CIT0037] Knight R L, and Rust M K 1990 Repellency and efficacy of insecticides against foraging workers in laboratory colonies of Argentine ants (Hymenoptera: Formicidae). J. Econ. Entomol. 83: 1402–1408.

[CIT0038] Krushelnycky P 2019 Evaluation of water-storing granules as a promising new baiting tool for the control of invasive ants in Hawaii. Report of Year 1 Activities to the Hawaii Invasive Species Council. Available from https://dlnr.hawaii.gov/hisc/files/2019/07/UH-CTAHR-KrushelnyckyP-Ant-Bait_FY18_Final-Report.pdf. Accessed 12 August 2020.

[CIT0039] Kulkarni A R, Soppimath K S, Aminabhavi T M, Dave A M, and Mehta M H 2000 Glutaraldehyde crosslinked sodium alginate beads containing liquid pesticide for soil application. J. Control. Release. 63: 97–105. 10640583 10.1016/s0168-3659(99)00176-5

[CIT0040] Kulkarni A R, Soppimath K S, Aminabhavi T M, and Dave A M 2002 Polymeric sodium alginate interpenetrating network beads for the controlled release of chlorpyrifos. J. Appl. Polym. Sci. 85: 911–918.

[CIT0041] Lao W, Tsukada D, Greenstein D J, Bay S M, and Maruya K A 2010 Analysis, occurrence, and toxic potential of pyrethroids, and fipronil in sediments from an urban estuary. Environ. Toxicol. Chem. 29: 843–851. 20821513 10.1002/etc.116

[CIT0042] Lee C C, Nakao H, Tseng S P, Hsu H W, Lin G L, Tay J W, Billen J, Ito F, Lee C Y, Lin C C, et al. 2017 Worker reproduction of the invasive yellow crazy ant *Anoplolepis gracilipes*. Front. Zool. 14: 24. 28503187 10.1186/s12983-017-0210-4 PMC5422973

[CIT0043] Leong J Y, Lam W H, Ho K W, Voo W P, Lee M F X, Lim H P, Lim S L, Tey B T, Poncelet D, and Chan E S 2016 Advances in fabricating spherical alginate hydrogels with controlled particle designs by ionotropic gelation as encapsulation systems. Particuology 24: 44–60.

[CIT0044] Markin G P, and Hill S O 1971 Microencapsulated oil bait for control of the imported fire ant. J. Econ. Entomol. 64: 193–196.

[CIT0045] Matulovic U, Rasch D, and Wagne F 1986 New equipment for the scaled up production of small spherical biocatalysts. Biotechnol. Lett. 8: 485–490.

[CIT0046] McCalla K, Tay J W, Mulchandani A, Choe D H, and Hoddle M 2020 Biodegradable alginate hydrogel bait delivery system effectively controls high-density populations of Argentine ant in commercial citrus. J. Pest Sci. 93: 1031–1042.

[CIT0047] Merrill K, Boser C L, Hanna C, Holway D A, Naughton I, Choe D H, and Wilson-Rankin E 2018 Argentine ant (*Linepithema humile*, Mayr) eradication efforts on San Clemente Island, California, USA. West. N. Am. Nat. 78: 829–836.

[CIT0048] Mihou A P, Michaelakis A, Krokos F D, Mazomenos B E, and Couladouros E A 2007 Prolonged slow release of (Z)-11-hexadecenyl acetate employing polyurea microcapsules. J. Appl. Entomol. 131: 128–133.

[CIT0049] Montesano F F, Parente A, Santamaria P, Sannino A, and Serio F 2015 Biodegradable superabsorbent hydrogel increases water retention properties of growing media and plant growth. Agric. Agric. Sci. Procedia 4: 451–458.

[CIT0050] Moreno D S, Haney P B, and Luck R F 1987 Chlorpyrifos and diazinon as barriers to Argentine ant (Hymenoptera, Formicidae) foraging on citrus trees. J. Econ. Entomol. 80: 208–214.

[CIT0051] Murata Y, Jinno D, Kofuji K, and Kawashima S 2004 Properties of calcium-induced gel beads prepared with alginate and hydrolysates. Chem. Pharm. Bull. (Tokyo). 52: 605–607. 15133215 10.1248/cpb.52.605

[CIT0052] (NRDC) Natural Resources Defense Council 2018 Hawaii bans use of toxic pesticide chlorpyrifos (updated 13 June 2018). Available from https://www.nrdc.org/experts/nrdc/hawaii-bans-use-toxic-pesticide-chlorpyrifos. Accessed 12 August 2020.

[CIT0053] Neiderud C J 2015 How urbanization affects the epidemiology of emerging infectious diseases. Infect. Ecol. Epidemiol. 5: 27060. 26112265 10.3402/iee.v5.27060 PMC4481042

[CIT0054] Nelson E H, and Daane K M 2007 Improving liquid bait programs for Argentine ant control: bait station density. Environ. Entomol. 36: 1475–1484. 18284776 10.1603/0046-225x(2007)36[1475:ilbpfa]2.0.co;2

[CIT0055] Oi D H, Vail K M, and Williams D F 2000 Bait distribution among multiple colonies of Pharaoh ants (hymenoptera: Formicidae). J. Econ. Entomol. 93: 1247–1255. 10985038 10.1603/0022-0493-93.4.1247

[CIT0056] Peck R W, Banko P C, Donmoyer K, Kropidlowski S, and Pollock A 2015 Efforts to eradicate yellow crazy ants on Johnston Atoll: results from crazy ant strike team IX, December 2014-June 2015. Technical Report HCSU-067, Hawaii Cooperative Studies Unit, University of Hawaii at Hilo, Hilo, HI

[CIT0057] Peck R W, Banko P C, Donmoyer K, Scheiner K, Karmi R, and Kropidlowski S 2017 Efforts to eradicate yellow crazy ants on Johnston Atoll: results from crazy ant strike team X, XI and XII, June 2015-December 2016. Technical Report HCSU-081, Hawaii Cooperative Studies Unit, University of Hawaii at Hilo, Hilo, HI.

[CIT0058] (PAN) Pesticide Action Network 2008 Which pesticides are banned in Europe? Available from https://www.pan-europe.info/old/Resources/Links/Banned_in_the_EU.pdf. Accessed 12 August 2020.

[CIT0059] Pimentel D 1995 Amounts of pesticides reaching target pests: environmental impacts and ethics. J. Agric. Environ. Ethic 8: 17–29.

[CIT0060] Raun E S, and Jackson R D 1966 Encapsulation as a technique for formulating microbial and chemical insecticides. J. Econ. Entomol. 59: 620–622.

[CIT0061] Richerson J V 1977 Pheromone-mediated behavior of the gypsy moth. J. Chem. Ecol. 3: 291–308.

[CIT0062] Roy A, Bajpai J, and Bajpai A K 2009a Dynamics of controlled release of chlorpyrifos from swelling and eroding biopolymeric microspheres of calcium alginate and starch. Carbohydr. Polym. 76: 222–231.

[CIT0063] Roy A, Bajpai A K, and Bajpai J 2009b Designing swellable beads of alginate and gelatin for controlled release of pesticide (cypermethrin). J. Macromol. Sci., Part A 46: 847–859.

[CIT0064] Roy A, Singh S K, Bajpai J, and Bajpai A K 2014 Controlled pesticide release from biodegradable polymers. Cent. Eur. J. Chem. 12: 453–469.

[CIT0065] Rudzinski W E, Dave A M, Vaishanav U H, Kumbar S G, Kulkarni A R, and Aminabhavi T M 2002 Hydrogels as controlled release devices in agriculture. Des. Monomers Polym. 5: 39–65.

[CIT0066] Rust M K, Reierson D A, and Klotz J H 2004 Delayed toxicity as a critical factor in the efficacy of aqueous baits for controlling Argentine ants (Hymenoptera: Formicidae). J. Econ. Entomol. 97: 1017–1024. 15279286 10.1093/jee/97.3.1017

[CIT0067] Rust M K, Choe D H, Wilson-Rankin E, Campbell K, Kabashima J, and Dimson M 2017 Controlling yellow jackets with fipronil-based protein baits in urban recreational areas. Int. J. Pest Manage. 63: 234–241.

[CIT0068] Rust M K, Soeprono A, Wright S, Greenberg L, Choe D H, Boser C L, Cory C, and Hanna C 2015 Laboratory and Field Evaluations of Polyacrylamide Hydrogel Baits Against Argentine Ants (Hymenoptera: Formicidae). J. Econ. Entomol. 108: 1228–1236. 26470250 10.1093/jee/tov044

[CIT0069] Saarai A, Kasparkova V, Sedlacek T, and Saha P 2011 A comparative study of crosslinked sodium alginate/gelatin hydrogels for wound dressing, pp. 384–389. *In* Mastorakis N (ed.), Proceeding of the 4th WSEAS International Conference on Engineering Mechanics, Structures, Engineering Geology. WSEAS Press, Greece.

[CIT0070] Sabadini R C, Martins V C A, and Pawlicka A 2015 Synthesis and characterization of gellan gum: chitosan biohydrogels for soil humidity control and fertilizer release. Cellulose 22: 2045–2054.

[CIT0071] Schall K, Tay J W, Mulchandani A, Choe D H, and Hoddle M 2018 Harnessing hydrogels in the battle against invasive ants: could a hydrogel baiting system solve Argentine ant problems in southern California citrus? Citrograph 9: 30–35.

[CIT0072] Scher H B 1977 Chapter 12 Microencapsulated pesticides, pp. 126–144. *In* Scher H B (ed.), Controlled release pesticides, vol. 53 ACS Symposium Series, American Chemical Society, Washington, DC.

[CIT0073] Sharma A, Kumar V, Shahzad B, Tanveer M, Sidhu G P S, Handa N, Kohli S K, Yadav P, Bali A S, Parihar R D, et al. 2019 Worldwide pesticide usage and its impacts on ecosystem. SN Appl. Sci. 1: 1446.

[CIT0074] Smidsrod O, and Draget K I 1996 Alginate gelation technologies, pp. 279–293. *In* Dickinson E and Bergenstahl B (eds.), Food colloids: proteins, lipids and polysaccharides. The Royal Society of Chemistry, Cambridge, United Kingdom.

[CIT0075] Stejskal V, Aulicky R, and Pekar S 2009 Brief exposure of *Blattella germanica* (Blattodea) to insecticides formulated in various microcapsule sizes and applied on porous and non-porous surfaces. Pest Manage. Sci. 65: 93–98. 10.1002/ps.1651 18823064

[CIT0076] Suiter D R, Schneider B M, Riegel C, Smith M S, and Bennett G W 2006 Brood reduction in noviflumuron-fed Pharaoh’s ant, *Monomorium pharaonis*, and Argentine ant, *Linepithema humile*, colonies (Hymenoptera: Formicidae). Sociobiology 47: 149–164.

[CIT0077] Swietoslawski J, Swietoslawski P, Liszka D, and Gliniewicz A 2011 Chapter 11 Encapsulation: an effective environmentally friendly technology for delivery of insecticides and repellents, pp. 156–168. *In* Dhang P (ed.), Urban pest management: an environmental perspective. CABI, Wallingford, United Kingdom.

[CIT0078] Tabaru Y, Mochizuki K, Watabe Y, and Takahashi T 2001 Repellency of insecticides against German cockroach, *Blattella germanica*, observed by feces distribution in insecticide-treated harborages. Med. Entomol. Zool. 52: 81–86.

[CIT0079] Tay J W, and Lee C Y 2014 Influences of pyriproxyfen on fecundity and reproduction of the Pharaoh ant (Hymenoptera: Formicidae). J. Econ. Entomol. 107: 1216–1223. 25026685 10.1603/ec14030

[CIT0080] Tay J W, and Lee C Y 2015a Induced Disturbances Cause Monomorium pharaonis (Hymenoptera: Formicidae) Nest Relocation. J. Econ. Entomol. 108: 1237–1242. 26470251 10.1093/jee/tov079

[CIT0081] Tay J W, and Lee C Y 2015b Effects of a juvenile hormone analogue pyriproxyfen on monogynous and polygynous colonies of the Pharaoh ant *Monomorium pharaonis* (Hymenoptera: Formicidae). Trop. Biomed. 32: 453–462. 26695205

[CIT0082] Tay J W, Neoh K B, and Lee C Y 2014 The roles of the queen, brood and worker castes in the colony growth dynamics of the pharaoh ant, *Monomorium pharaonis*(Hymenoptera: Formicidae). Myrmecol. News 20: 87–94.

[CIT0083] Tay J W, Hoddle M S, Mulchandani A, and Choe D H 2017 Development of an alginate hydrogel to deliver aqueous bait for pest ant management. Pest Manag. Sci. 73: 2028–2038. 28517237 10.1002/ps.4616

[CIT0084] Tønnesen H H, and Karlsen J 2002 Alginate in drug delivery systems. Drug Dev. Ind. Pharm. 28: 621–630. 12149954 10.1081/ddc-120003853

[CIT0085] Tsuji K 2001 Microencapsulation of pesticides and their improved handling safety. J. Microencapsul. 18: 137–147. 11253931 10.1080/026520401750063856

[CIT0086] (US EPA) U. S. Environmental Protection Agency 2002 Interim registration eligibility decision for chlorpyrifos. EPA 738-R-01-007. Available from https://nepis.epa.gov/Exe/ZyPDF.cgi/200008BM.PDF?Dockey=200008BM.PDF. Accessed 12 August 2020.

[CIT0087] Wang Q, Xie X, Zhang X, Zhang J, and Wang A 2010 Preparation and swelling properties of pH-sensitive composite hydrogel beads based on chitosan-g-poly (acrylic acid)/vermiculite and sodium alginate for diclofenac controlled release. Int. J. Biol. Macromol. 46: 356–362. 20096301 10.1016/j.ijbiomac.2010.01.009

[CIT0088] Welzel K F, and Choe D H 2016 Development of a Pheromone-Assisted Baiting Technique for Argentine Ants (Hymenoptera: Formicidae). J. Econ. Entomol. 109: 1303–1309. 26912774 10.1093/jee/tow015

[CIT0089] Weston D P, Holmes R W, and Lydy M J 2009 Residential runoff as a source of pyrethroid pesticides to urban creeks. Environ. Pollut. 157: 287–294. 18676072 10.1016/j.envpol.2008.06.037

[CIT0090] (WHO) World Health Organization 1985 International programme on chemical safety—environmental health criteria 49: acrylamide. WHO, Geneva Available from http://www.inchem.org/documents/ehc/ehc/ehc49.htm. Accessed 12 August 2020.

[CIT0091] Zhu T, Mao J, Cheng Y, Liu H, Lv L, Ge M, Li S, Huang J, Chen Z, Li H, Yang L, and Lai Y 2019 Recent progress of polysaccharide-based hydrogel interfaces for wound healing and tissue engineering. Adv. Mater. Interfaces 6: 1900761.

